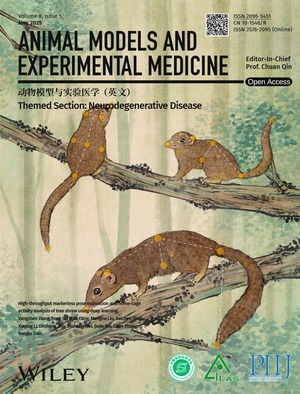# Cover Picture

**DOI:** 10.1002/ame2.12430

**Published:** 2025-05-27

## Abstract

This cover image is based on the article “High‐throughput markerless pose estimation and home‐cage activity analysis of tree shrew using deep learning” reported by Yangzhen Wang, Feng Su, Rixu Cong, Mengna Liu, Kaichen Shan, Xiaying Li, Desheng Zhu, Yusheng Wei, Jiejie Dai, Chen Zhang, Yonglu Tian (https://doi.org/10.1002/ame2.12530). This study presents a deep learning approach that achieves markerless pose estimation and recognizes diverse natural behaviors in tree shrews. By capturing detailed body movements with high precision, this method offers an efficient and cost‐effective solution for studying tree shrews. The system enables high‐throughput, long‐term behavioral monitoring, making it a powerful tool for behavioral research. It offers new insights into their behavioral patterns and daily routines, deepening our understanding of their complex behaviors.